# Sex differences in physical functioning among older adults: cross-sectional results from the OUTDOOR ACTIVE study

**DOI:** 10.1186/s12889-024-19218-x

**Published:** 2024-07-02

**Authors:** Imke Stalling, Martin Gruber, Karin Bammann

**Affiliations:** Institute of Public Health and Nursing Research (IPP), University of Bremen, Grazer Straße 2a, 28359 Bremen, Germany

**Keywords:** Physical functional performance, Aged, Sex differences, Healthy ageing

## Abstract

**Background:**

Maintaining good functional ability is a key component of healthy ageing and a basic requirement for carrying out activities of daily living, staying independent, and delaying admission to a nursing home. Even though women have a higher life expectancy and slower age-related muscle mass loss than men, they often show a higher prevalence of limitations in physical functioning. However, the reasons behind these sex differences are still unclear. Therefore, the aims of this study were to investigate sex differences among older adults regarding physical functioning and to study which factors are explaining these sex differences.

**Methods:**

Cross-sectional data from participants of the OUTDOOR ACTIVE study residing in Bremen, Germany, aged 65 to 75 years, were included in the analyses. Physical functioning was assessed via a self-administered questionnaire using the SF-36 10-item Physical Functioning Scale. Social, lifestyle, and health-related factors were also assessed using the questionnaire. Physical activity was measured objectively using wrist-worn accelerometers over seven consecutive days. Descriptive analyses with absolute and relative frequencies, means and standard deviations, as well as T-tests and chi-square tests were carried out. To test for associations between sex, physical functioning, and several individual factors, linear regressions were performed.

**Results:**

Data of 2 141 participants (52.1% female) were included in the study. Women and men showed statistically significant differences in physical functioning, with men perceiving fewer limitations than women. On average, women had a physical functioning score of 81.4 ± 19.3 and men 86.7 ± 17.0. Linear regression showed a statistically significant negative association between physical functioning score and sex (β: -0.15, 95% CL: -0.19, -0.10). The association remained statistically significant when adding individual factors to the model. All factors together were only able to explain 51% of the physical functioning-sex association with health indicators and the presence of chronic diseases being the most influential factors.

**Conclusions:**

We found sex differences in physical functioning, with older women having more limitations than older men. The results showed that health-related factors and chronic diseases played the biggest roles in the different physical functioning scores of women and men. These findings contribute to future longitudinal, more in-depth research.

**Trial registration:**

German Clinical Trials Register DRKS00015117 (Date of registration 17-07-2018).

**Supplementary Information:**

The online version contains supplementary material available at 10.1186/s12889-024-19218-x.

## Background

Maintaining functional ability into higher age is a key component of healthy ageing and has profound consequences on the individual, societal, and economic levels [1, 2]. Good physical functioning is a basic requirement for carrying out activities of daily living (ADL), staying independent, and delaying admission to a nursing home [3]. These are important goals not only for older adults but also for the health system [4].

Physical functioning declines with age, which in most cases leads to frailty and disability [5]. Furthermore, low physical functioning is associated with decreased quality of life [6], an increased risk of hospitalisation [7, 8] and mortality [9, 10]. It is believed, that the decrease of muscle mass, which usually starts during midlife, plays an important role in diminishing physical functioning [11]. The extent to which other factors influence the decline of physical functioning has not been fully investigated, but previous cross-sectional studies have found associations between physical functioning and social support [12], socioeconomic status [13], nutrition [14], and body mass index (BMI) [15]. Longitudinal research has also found physical functioning to be affected by BMI [16], as well as social isolation [17] and physical activity [18].

Even though women have a higher life expectancy, lower risks of several non-communicable diseases, and slower age-related muscle mass loss than men [19–21], they often show a higher prevalence of limitations in physical functioning. Hansen et al. [22] for example, found in their sample of 60 to 70-year-old Danes, that 16.8% of men and 20.4% of women displayed limitations in at least two out of three activities. Furthermore, women experience a faster decline in physical functioning and live longer with disabilities [23, 24]. Sex differences regarding limitations in physical functioning are already present among younger adults and broaden with increasing age [25]. Sialino et al. found in their Dutch study an average difference in the physical functioning score (possible 0 to 100 points) of 6 points at the age of 45 and 12 points at age 80, with women scoring lower points than men [25]. However, the reasons behind women having a higher prevalence of limitations in physical functioning are still unclear [11]. First studies have found work and family characteristics [24] as explanations for sex differences in physical functioning among working Japanese adults. Since health behaviours, biological factors, and reporting behaviour (i.e., willingness to report symptoms, recollection of minor health problems) are associated with sex differences in overall health [26–28], they are being discussed as potential explanations for sex differences in physical functioning [11, 25, 29].

Understanding the influences of physical functioning in women and men can help designing health-related interventions to maintain physical ability. Since women tend to get older and live alone for a longer period, it is important to investigate the reasons behind the physical functioning differences. Therefore, the study aims to (a) investigate sex differences among older adults regarding physical functioning and (b) to study whether social, lifestyle, and health-related factors are predicting these sex differences.

## Methods

### Study design and sample

This cross-sectional study is based on the OUTDOOR ACTIVE project, which was part of the prevention research network AEQUIPA in north-western Germany [30]. The project aimed to assess physical activity (PA) among older adults between 65 and 75 years and identify drivers and barriers of PA. Additionally, a community-based physical activity program for older adults was developed using participatory methods. The OUTDOOR ACTIVE study was divided into a pilot study (02/2015 to 01/2018) and a registered cluster-randomized controlled trial (c-RCT) (02/2018-12/2022) [31].

#### Participants and recruitment

Eligibility criteria included living in pre-defined subdistricts of Bremen (pilot study: Arbergen, Hastedt, Hemelingen, Mahndorf, Sebaldsbrueck; c-RCT: Blumenthal, Burg-Grambke, Gete, Lehe, Lehesterdeich, Neustadt, Ohlenhof, Ostertor), being between 65 and 75 years old, and not being institutionalised. Exclusion criteria were moving out of the study region, language barriers, acute health problems (i.e., every illness or injury that prevented participation), and death. Address data were obtained from the Registration Office Bremen, which is authorized for scientific research. Initially, all potential participants (*n* = 10 928) were sent an invitation letter and later contacted by phone. Of these, 3 425 individuals were never reached, 4 247 refused participation, and 1 115 were excluded.

The ethics committee of the University of Bremen approved both study parts and all participants provided written informed consent.

### Measures

Both the pilot study and the c-RCT consisted of a baseline survey and a follow-up survey, which comprised a self-administered paper-pencil questionnaire, a short physical examination followed by a fitness test, and seven-day accelerometry to objectively measure PA [31, 32]. All assessments were carried out by trained members of the study team, usually a research assistant and a student assistant. To ensure the assessments were done as standardized as possible and therefore minimizing the risks of potential biases, a survey manual was developed and handed to each member of the study team in addition to the training. Additionally, regular quality checks during the survey were carried out.

#### Social factors

Information on age, sex, marital status, and having a partner were assessed using a self-administered questionnaire. For socioeconomic status (SES), an additive social class index was calculated, consisting of education, income, and occupation (for more details see [32]), with a possible maximum of 100 points. Social support was assessed using the Oslo Social Support Scale [33] and calculating a score (3–14 points), with higher results indicating better social support. Using a modified question from the German Health Interview and Examination Survey [34] participants were asked if they lived alone and, if not, to state their household size (“*How many people live in your household?*”, response categories: “*I live alone*”, “*We are _ people in the household”*).

#### Physical functioning

Physical functioning was assessed via a self-administered paper-pencil questionnaire using the 10-item Physical Functioning Scale (items 3a-3j) from the MOS 36-Item Short Form Health Survey (Version 1.0) (SF-36) [35]. Participants were asked if they had any limitations regarding ten different activities. Possible answers were *limited a lot, limited a little*, and *not limited at all*. A score was calculated with a possible range of 0 to 100 points. Higher scores indicate better physical functioning.

#### Health-related factors

Self-rated health status and bodily pains in the past four weeks were assessed using questions from the SF-36 (items 1 and 7) [35]. A modified question from the German Health Interview and Examination Survey [34] on chronic diseases was used (“*Do you have one or more long-term, chronic diseases?*”). A list of chronic diseases was provided, where participants checked the ones they had. Using a self-developed question, participants were asked if they take medications daily (“*Do you take medications daily?*”) with the response categories “*No*” and “*Yes, namely*” followed by an open text field. Shortness of breath during light PA was assessed using a self-developed question (“*Are you short of breath during light exertion, e.g. during short walks, light gardening or after climbing a few steps?*”), with the response categories “*Yes*” and “*No*”.

During the short physical examination body weight and height were measured using a Kern MPC 250K100M personal floor scale (Kern & Sohn GmbH, Balingen, Germany) and a Seca 217 mobile stadiometer (Seca GmbH & Co. KG, Hamburg, Germany), respectively. Subsequently, body mass index (BMI) was calculated by dividing body weight (in kg) by the squared height (in m). The classification of underweight (< 18.5 kg/m^2^), normal weight (18.5 kg/m^2^-24.9 kg/m^2^), overweight (25 kg/m^2^-29.9 kg/m^2^), and obesity (≥ 30 kg/m^2^) by the World Health Organization was used [36].

The consumption of alcohol was assessed using a self-developed question on food frequency (“*How often do you eat or drink the following foods?*”) with six response categories (“*never*”, “*once a month or less*”, “*two to three times a month*”, “*once a week*”, “*several times a week*”, “*(almost) daily*”). In the questionnaire, more food items were included, but for this analysis, only alcohol as a potential health-compromising behaviour was included. For the analyses, response categories of “*never*” to “*several times a week*” were scored as 0 and “*(almost) daily*” as 1.

#### Physical activity

Physical activity was measured objectively and via self-report. For objective measurement, the ActiGraph GT3x-BTw (ActiGraph LLC, Pensacola, FL, USA) accelerometers were used. Acceleration and deceleration of the body are measured in three axes [37]. Sampling frequency was set to 30 Hz, data were downloaded and processed using ActiLife (Version 6.13.3 ActiGraph LLC, Pensacola, FL, USA). Participants were asked to wear the devices for seven consecutive days on their non-dominant wrist, if possible, for 24 h. Vector magnitude counts were calculated from the data of the three axes and non-wear time was defined as 90 consecutive minutes with zero counts [38]. Average daily counts per minute (CPM) reflect the total amount of PA and were included in the analyses. Active transport was assessed using a question based on the Neighbourhood Environment and Walkability Scale (NEWS) [39] using 12 common destinations. Participants stated their usual mode of transportation and the minutes spent on one trip (for further details see [40]). Transport via bike or on foot was combined for active transport for the present study.

### Statistical analyses

The statistical analyses only comprise data from the baseline survey. Descriptive analyses with absolute and relative frequencies were carried out for sex, marital status, having a partner, SES, self-rated health, BMI, items of the physical functioning score, and the categories of physical functioning. Means and standard deviations were calculated for age and physical functioning score. T-tests and chi-square tests were carried out to test for statistically significant differences between women and men regarding the physical functioning score and physical functioning items.

To test whether the association between sex and physical functioning can be explained by other individual factors, we standardized the data using the SAS procedure PROC STDIZE (SAS Institute, Cary (NC), USA), where ${z}_{i}$ is the standardized value of ${x}_{i}$ given ${(x}_{i}-\bar{x})/s$ with $\bar{x}$ being the variables’ mean and $s$ the standard deviation. Then, the following approach was pursued: Firstly, we grouped the individual factors into the following groups: vertical social factors, horizontal social factors, lifestyle factors, health indicators and (presence of) chronic diseases. Secondly, we performed ordinary least squares (OLS) regressions (complete case analyses) with physical functioning as dependent and the individual factors and age as independent variables to check whether the variable has an association with physical functioning. Thirdly, for each group of individual factors with at least one statistically significant association with physical functioning, we performed an OLS regression (complete cases) with physical functioning as dependent and the statistically significant individual factors of that group and sex and age as independent variables to check if and to what extent the association between sex and physical functioning can be explained by including these individual factors. For the latter, we (a) performed an OLS regression with physical functioning as dependent and sex as independent variable and saved the residuals, and (b) performed OLS regressions (complete cases) with the calculated residuals as dependent and the individual factors of each group and age as the independent variables. For this last step, we reported the explained variance of the residuals R^2^. For all other OLS regressions we reported the standardized beta β with 95%-confidence limits. The number of missing data for each individual factor is being displayed in Additional file 1. A sensitivity analysis excluding participants with missing data was conducted. To check violations of regression model assumptions, studentized residuals were calculated for each of the models. Except for the standardization of variables, all statistical analyses were conducted with SPSS 22.0 (IBM Corp. Armonk (NY), USA). The threshold for statistical significance was set at *p* < 0.05.

## Results

The characteristics of the 2 141 participants of both the pilot study and the c-RCT are displayed in Table 1. 52.1% of the participants were female and the mean age was 69.8 ± 3.0 years. The majority was married and had a partner. Women were mostly in the lower SES quintiles, while most of the men pertained to the higher SES quintiles. Both women and men mostly rated their health as good. Most participants were either overweight or obese.

**Table 1:** Characteristics of the study population

	All survey participants (*n* = 2 141)	
	Women (*n* = 1 115)	Men (*n* = 1 026)
	Mean (SD)	
Age (years)	69.8 (3.0)	69.8 (3.0)
	*n* (%)	
Marital status		
Married	590 (55.3)	766 (78.0)
Divorced	192 (18.0)	101 (10.3)
Widowed	190 (17.8)	47 (4.8)
Single/unwed	94 (8.8)	68 (6.9)
Having a partner^§^	663 (63.7)	840 (87.7)
Socioeconomic status (SES)		
1st quintile (lowest)	256 (23.7)	160 (16.1)
2nd quintile	249 (23.1)	164 (16.5)
3rd quintile	206 (19.1)	206 (20.8)
4th quintile	190 (17.6)	226 (22.8)
5th quintile (highest)	178 (16.5)	235 (23.7)
Self-rated health		
Less good or bad	196 (18.4)	159 (16.2)
Good	620 (58.2)	546 (55.6)
Very good or excellent	250 (23.5)	277 (28.2)
Body mass index (BMI)^$^		
Underweight	9 (1.0)	0 (0.0)
Normal weight	372 (41.6)	214 (27.0)
Overweight	329 (36.8)	402 (50.7)
Obesity	184 (20.6)	177 (22.3)

SD: Standard deviation

^§^ Every participant, who has stated that they have a partner is in this category, regardless of marital status.

^$^ Classification of BMI: underweight: <18.5 kg/m^2^, normal weight: 18.5 kg/m^2^-24.9 kg/m^2^, overweight: 25 kg/m^2^-29.9 kg/m^2^, obesity: ≥30 kg/m^2^

Detailed information on physical functioning is shown in Table 2. Women and men showed statistically significant differences in nine out of ten physical functioning items, with men perceiving fewer limitations than women. Only the item *bathing or dressing oneself* showed no significant differences. The majority of the participants had no limitations when walking one hundred metres, bathing or dressing oneself, climbing one flight of stairs, and walking several hundred metres. *Vigorous activities* was the item where most participants showed limitations. Most limitations were reported regarding carrying out vigorous activities. The distribution of physical functioning categories showed statistically significant sex differences, with men having higher scores than women.

**Table 2:** Differences in physical functioning between women and men

	Women (*n* = 1 115)	Men (*n* = 1 026)	Test of significance
	Mean (SD)		t
Physical functioning score^§^	81.4 (19.3)	86.7 (17.0)	6.4***
	*n* (%)		χ^2^
No limitations regarding…			
…vigorous activities	231 (22.1)	299 (31.1)	23.0***
…moderate activities	698 (66.3)	768 (80.1)	48.3***
…lifting or carrying groceries	639 (60.7)	820 (85.1)	150.6***
…climbing several flights of stairs	621 (59.2)	697 (72.8)	42.7***
…climbing one flight of stairs	910 (87.9)	874 (91.5)	7.1*
…bending, kneeling, or stooping	537 (50.9)	554 (57.5)	15.2**
…walking more than one kilometre	771 (73.6)	776 (80.3)	13.3**
…walking several hundred metres	888 (84.6)	867 (90.1)	14.5**
…walking one hundred metres	948 (90.2)	900 (94.1)	10.7**
…bathing or dressing self	958 (91.2)	882 (92.0)	0.5

^§^Scores range from 0 = highest physical impairment to 100 = no physical impairment

* *p*-value < 0.05

** *p*-value < 0.01

*** *p*-value < 0.001

**Fig. 1:**
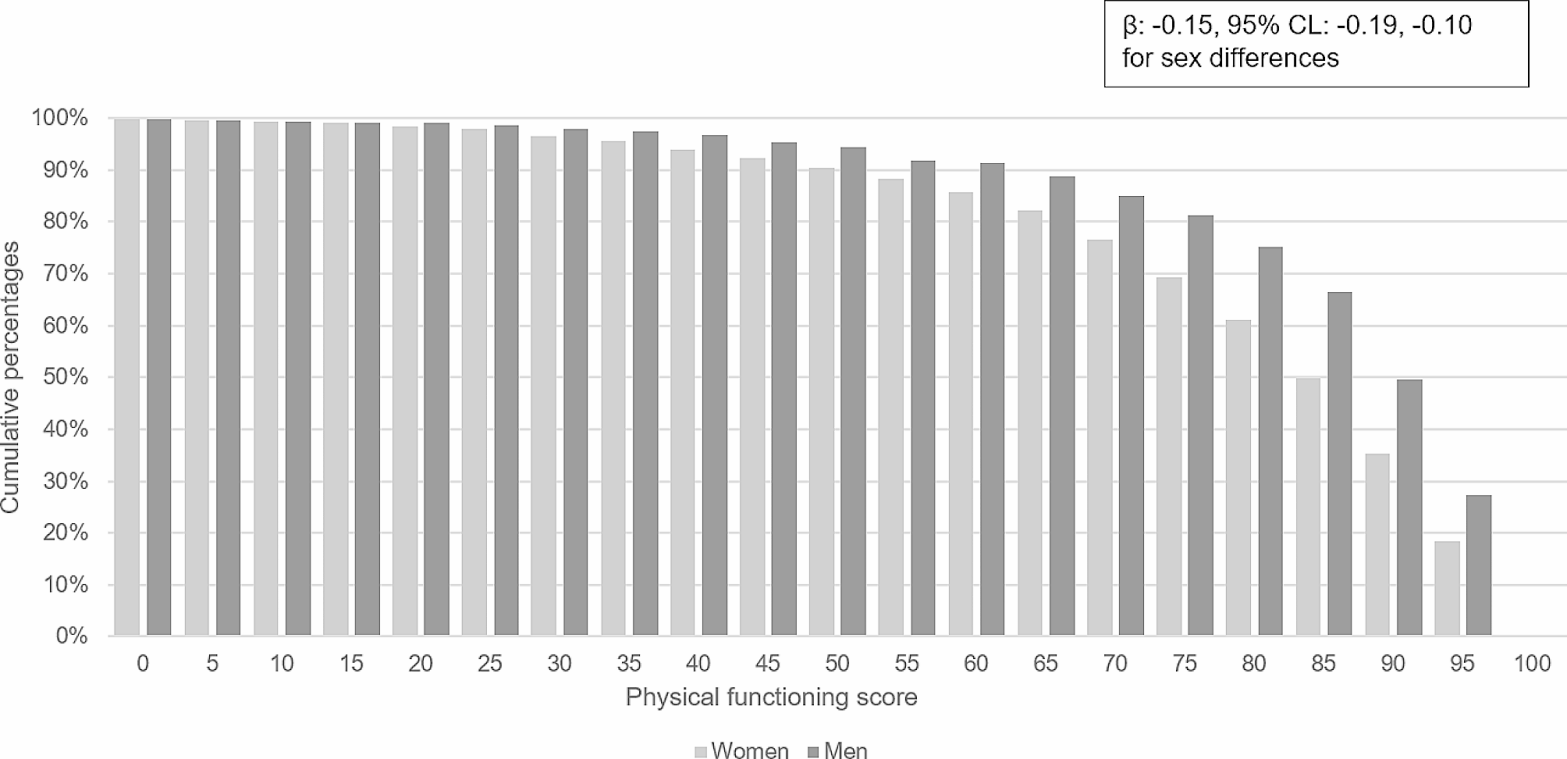
Sex differences in physical functioning score, cumulative percentages

Figure 1 shows the cumulative percent of participants reaching the different physical functioning scores. For example, 90.3% of women and 94.3% of men scored at least 50 points in physical functioning, while 35.3% of women and 49.6% of men scored at least 90 points. Linear regression showed a statistically significant negative association between physical functioning score and sex (β: -0.15, 95% CL: -0.19, -0.10).

Table 3 shows the results of the analyses which individual factors were able to predict the sex-physical functioning association. Linear regression results showed that a higher physical functioning score was statistically significantly positively associated with having a higher SES, having a partner, higher total amount of PA, higher amount of active transport per week, and drinking alcohol daily. Statistically significant negative associations with physical functioning were seen for poor social support, living alone, all health indicators, and all chronic diseases. The association between sex and physical functioning remained statistically significant when adding SES, horizontal social factors (i.e., poor social support, having a partner, living alone), lifestyle factors, health indicators, and chronic diseases. All factors together were only able to predict 51% of the physical functioning-sex association, with health indicators (R^2^: 0.46) and the presence of chronic diseases (R^2^: 0.22) being the most influential factors.

**Table 3:** Explanatory factors for the sex-physical functioning association, linear regression, age-adjusted, *n* = 2 141

	Women	Men	Individual factors^§^ on physical functioning	Sex on physical functioning	*R*^2^ for sex – physical functioning residuals
	*n* = 1 115	*n* = 1 026	β (95% CL)	β (95% CL)	
*Vertical social factors*					
Socioeconomic status (Mean, (SD))	58.1 (12.9)	62.1 (13.5)	0.23 (0.19, 0.28)***	-0.11 (-0.16, -0.07)***	0.06
*Horizontal social factors*				-0.14 (-0.19, -0.09)***	0.04
Poor social support	8.8%	11.6%	-0.13 (-0.18, -0.08)***		
Having a partner	63.7%	87.7%	0.08 (0.04, 0.13)***		
Living alone	39.7%	18.1%	-0.07 (-0.11, -0.02)**		
*Lifestyle factors*				-0.22 (-0.25, -0.15)***	0.06
Physical activity (Average VM CPM (SD))	1830.8 (501.2)	1516.0 (424.7)	0.12 (0.07, 0.16)***		
Active transport (Average min/week (SD))	75.8 (56.7)	66.6 (57.3)	0.05 (0.00, 0.09)*		
Daily alcohol consumption	7.0%	17.3%	0.10 (0.05, 0.14)***		
*Health indicators*				-0.12 (-0.14, -0.07)***	0.46
Poor self-rated health	18.4%	16.2%	-0.59 (-0.64, -0.57)***		
Overweight or obesity	57.4%	73.0%	-0.17 (-0.20, -0.11)***		
Bodily pain (last 4 weeks)	73.3%	67.6%	-0.32 (-0.36, -0.28)***		
Shortness of breath	21.2%	14.7%	-0.50 (-0.55, -0.47)***		
*Chronic diseases*				-0.10 (-0.14, -0.06)***	0.22
Arthrosis, arthritis	42.6%	21.7%	-0.31 (-0.35, -0.26)***		
Incontinence	15.1%	11.8%	-0.16 (-0.20, -0.11)***		
Depression	5.1%	3.1%	-0.14 (-0.18, -0.10)***		
Diabetes mellitus	6.8%	11.3%	-0.14 (-0.18, -0.09)***		
Heart diseases	7.3%	18.4%	-0.17 (-0.22, -0.13)***		
Osteoporosis	10.7%	2.0%	-0.16 (-0.21, -0.12)***		
Rheumatism	6.1%	3.3%	-0.23 (-0.28, -0.19)***		
Hearing impairment	9.8%	14.1%	-0.08 (-0.13, -0.04)***		
All variables	-	-	-	-0.10 (-0.14, -0.05)***	0.51

COPD: Chronic obstructive pulmonary disease

VM CPM: Vector Magnitude counts per minute

β: Standardized Beta

CL: Confidence level

^§^ Only variables that were significantly associated with physical functioning and sex were included. The following variables were not included: number of children, frequency of seeing family, member in a sports club, daily medication intake, allergies, COPD, cancer, Parkinson’s disease

* *p*-value < 0.05

** *p*-value < 0.01

*** *p*-value < 0.001

## Discussion

This study investigated sex differences among older adults regarding physical functioning and whether social, lifestyle, and health-related factors predicted these. Results showed sex differences regarding physical functioning, with men perceiving fewer limitations than women. The statistically significant negative associations between physical functioning and sex remained after adding vertical social factors, horizontal social factors, lifestyle factors, health indicators, and chronic diseases, both individually and all together in a model. The physical functioning-sex association depended mostly on health indicators (R^2^: 0.46), followed by chronic diseases (R^2^: 0.22).

Our results regarding sex differences in physical functioning are in line with previous research, which found women to perceive more limitations and therefore a lower physical functioning score than men [24, 25, 41, 42]. Sialino et al. found a difference in physical functioning scores of 6.55 points on average [25] and von Bonsdorff et al. [43] found a mean difference of 6.67 points, with women having lower scores. While our results showed a slightly smaller difference of 5.3 points, the findings strengthen the evidence of sex differences regarding physical functioning, especially for German older adults.

We found SES to be statistically significantly positively associated with physical functioning and negatively with sex. However, SES predicted only little of the association between sex and physical functioning (R^2^: 0.06). Previous research found contrasting results. While Hansen et al. [44] found a statistically significant negative association between social class and physical functioning, they found no differences regarding this association between men and women. Their study sample, however, consisted of middle-aged adults between 50 and 60 years and they measured physical functioning objectively with a physical performance test. Park et al. [27], on the other hand, found SES to have an impact on the sex differences in physical functioning among older adults, although they defined physical functioning as having limitations in ADL (i.e., basic needs such as eating, getting dressed, hygiene [45]) and instrumental ADL (IADL, i.e., shopping, doing housework, financial tasks [46]). Pajak et al. [47] also found SES to be statistically significantly positively associated with physical functioning in their study of the 45 to 65-year-old Polish population. They also used the Physical Functioning Scale from SF-36 and showed that the social gradient regarding physical functioning was larger in women than in men.

Our results indicated that health indicators have one of the highest influences on sex differences in physical functioning. Though the sex difference remained statistically significant, health indicators decreased the association most. These findings are in line with previous research. Kuh et al. [41] found women to have poorer overall health, which leads to weakness and therefore worse physical functioning. Although that study only included participants aged 53 years and were therefore younger than our study sample, these findings can be applied to older women as well, since muscle mass and strength tend to start declining around midlife [11, 48]. Further, previous research showed bodily pain to be an important determinant of physical functioning. In the study by Sialino et al. [25], the intensity of bodily pain was reported as more severe among women and was associated with decreased physical functioning. They further found BMI to be negatively associated with physical functioning, however, a higher BMI was more prevalent among men. These findings could also be observed in our results, indicating that overweight and obesity should be considered when trying to improve physical functioning among older adults, especially men.

When looking at chronic diseases, the association between sex and physical functioning decreased but remained statistically significant. Sialino and colleagues [25] found chronic diseases to be one of the strongest determinants associated with physical functioning. Additionally, previous studies have shown that physical functioning decreases with an increasing number of chronic diseases [49, 50]. Especially chronic diseases, that can lead to disabilities, predicted significantly more problems and functional limitations, such as Parkinson’s disease, arthritis, past stroke, and kidney stones in the study by Koukouli et al. [12]. While some evidence suggests that men display higher prevalences of chronic diseases [51], previous research found that women more often suffer from disabling and non-lethal diseases, such as arthritis or depression, which can lead to limitations in physical functioning [52, 53].

In our study, lifestyle factors and horizontal social factors, such as poor social support, having a partner, and living alone, did not explain sex differences regarding physical functioning. Previous studies have shown higher physical activity to be related to better physical functioning among older adults [42, 54]. While in the study by Mosallanez and colleagues [42] women were less physically active and showed lower levels of physical functioning than men, our results differed with women being more physically active, but still showing lower physical functioning scores. A factor explaining these differences might be that we used objective measures of PA and the Swedish study used subjective measures. In our study, accelerometers were worn on the non-dominant wrist, which can detect more upper body movement than other placements, like the hip [55]. In a previous publication, we found that the women devoted more time than the men in our study sample to household activities, which include a lot of upper body movements [40]. This could lead to higher PA levels of women. Furthermore, when interpreting the subjective measures used in the Swedish study misreporting of PA in questionnaires has to be taken into account. Dyrstad et al. [56] found that men tend to report higher PA levels than women.

Câmara et al. [57] found associations between social interactions and better physical performance while living alone was associated with worse physical functioning in men but not in women. Previous research has shown that loneliness is a risk factor for frailty [58] and disability [59], which both result in lower physical functioning. Our results also indicated that living alone is associated with worse physical functioning.

Other possible explanations for sex differences in physical functioning have been discussed in the literature. One main topic is biological causes for different physical functioning among older adults. It is assumed that menopause can lead to loss of muscle mass, which in turn can lead to lower muscle strength compared to men [48]. Another possible explanation is a difference in response behaviour. There have been discussions that women tend to report more disability and limitations than men because they perceive their physical functioning to be dysfunctional more frequently [60]. Additionally, it is assumed that men are more likely to neglect pain and disease, because of social conditioning [60]. However, previous studies have found women’s physical functioning to also be more limited than men’s when using objective measures, such as fitness tests [61, 62]. Furthermore, when discussing physical functioning of older adults, it is important to take into account, that physical functioning seems to be a dynamic process that can be improved even when limitations have been reported [63].

When comparing research on physical functioning, the different understandings of what this term includes and how it is being measured pose a problem. The most common measures are assessing limitations in ADL or IADL [27, 64–66], conducting various fitness tests [11, 54, 57, 61, 62], or, as we did in our study, using the SF-36 physical functioning scale [18, 24, 25, 67, 68]. While there is some overlap in the assessment of the different measures, they could come to varying conclusions.

This study has some strengths and limitations that need to be discussed. Firstly, the analyses were only implemented cross-sectionally; therefore, no statements regarding causation can be made. Furthermore, we do not have any information on the mentioned biological factors, such as menopause and the resulting loss of muscle mass, which could be the main factors for sex differences in physical functioning. Since most of the variables used were self-reported data, the results have to be interpreted with caution. Social desirability, over- and underreporting could potentially distort the results and should be taken into account. A selection bias is possible, since participants could choose in which parts of the study they wanted to partake in. Furthermore, the data used in this study stem from older adults residing in Bremen, Germany, which is why the results cannot be generalized for all older adults. However, the findings give an indication which factors play a role in sex differences regarding physical functioning. The strength of this study is that we included a wide range of different possible factors. In many studies that investigated physical functioning, sex differences were found, but they were not further explored. The studies that investigated sex differences mostly focused on one specific dimension, such as social factors, but only very few included a variety of possible factors.

## Conclusion

This study found sex differences regarding physical functioning among older adults, with women having more limitations than men. The results showed that health factors, such as poor self-rated health, overweight or obesity, pains, and shortness of breath as well as chronic diseases can predict sex differences regarding physical functioning of older adults. Longitudinal research and a more distinct definition of physical functioning and its measures are needed to get a better understanding of sex differences in physical functioning.

### Electronic supplementary material

Below is the link to the electronic supplementary material.

Supplementary Material 1

## Data Availability

The datasets used and/or analysed during the current study are available from the corresponding author on reasonable request.

## Abbreviations

ADL: Activities of daily living
BMI: Body mass index
c-RCT: Cluster-randomized controlled trial
CL: Confidence limits
COPD: Chronic obstructive pulmonary disease
IADL: Instrumental activities of daily living
PA: Physical activity
SES: Socioeconomic status
