# Supplementary material for: Sex differences in physical functioning among older adults: cross-sectional results from the OUTDOOR ACTIVE study
Source: BMC Public Health. 2024 Jul 2;24:1766. doi: 10.1186/s12889-024-19218-x (PMC11221023; doi:10.1186/s12889-024-19218-x)
Supplement: Supplementary file 1 — Supplementary Material 1 [file 12889_2024_19218_MOESM1_ESM.docx]

**Additional file 1 Missing data for variables used in Table 3**

|  | Women | Men |
| --- | --- | --- |
|  | n=1115 | n=1026 |
|  | n (%) | |
| *Vertical social factors* |  |  |
| Socioeconomic status | 36 (3.2) | 34 (3.3) |
|  |  |  |
| *Horizontal social factors* |  |  |
| Poor social support | 60 (5.4) | 62 (6.0) |
| Having a partner | 75 (6.7) | 68 (6.6) |
| Living alone | 38 (3.4) | 39 (3.8) |
|  |  |  |
| *Lifestyle factors* |  |  |
| Physical activity (VM CPM) | 316 (28.3) | 303 (29.5) |
| Active transport (min/week) | 145 (13.0) | 141 (13.7) |
| Daily alcohol consumption | 52 (4.7) | 45 (4.4) |
|  |  |  |
| *Health indicators* |  |  |
| Poor self-rated health | 49 (4.4) | 44 (4.3) |
| Overweight or obesity | 221 (19.8) | 233 (22.7) |
| Bodily pains (last 4 weeks) | 49 (4.4) | 54 (5.3) |
| Shortness of breath | 56 (5.0) | 49 (4.8) |
|  |  |  |
| *Chronic diseases* |  |  |
| Arthrosis, arthritis | 41 (3.7) | 48 (4.7) |
| Incontinence | 41 (3.7) | 48 (4.7) |
| Depression | 41 (3.7) | 48 (4.7) |
| Diabetes mellitus | 41 (3.7) | 48 (4.7) |
| Heart diseases | 41 (3.7) | 48 (4.7) |
| Osteoporosis | 41 (3.7) | 48 (4.7) |
| Rheumatism | 41 (3.7) | 48 (4.7) |
| Hearing impairment | 41 (3.7) | 48 (4.7) |
